# Response of Brain Metastases From *PIK3CA*-Mutant Breast Cancer to Alpelisib

**DOI:** 10.1200/PO.19.00403

**Published:** 2020-05-27

**Authors:** Felipe Batalini, Stacy L. Moulder, Eric P. Winer, Hope S. Rugo, Nancy U. Lin, Gerburg M. Wulf

**Affiliations:** ^1^ Beth Israel Deaconess Medical Center, Boston, MA; ^2^ MD Anderson Cancer Center, Houston, TX; ^3^ Dana-Farber Cancer Institute, Boston, MA; ^4^ University of California, San Francisco, San Francisco, CA

## INTRODUCTION

Activating mutations of *PIK3CA* are found in 25%-40% of estrogen receptor–positive (ER+), HER2-negative (HER2−) breast cancers (BC), and in 8% of ER-negative (ER−) BC.^1-5^ Two recent studies support the benefit of PI3K inhibition in combination with endocrine therapy. SANDPIPER (study of taselisib plus fulvestrant *v* placebo plus fulvestrant in participants with advanced or metastatic breast cancer who have disease recurrence or progression during or after aromatase inhibitor therapy) demonstrated a small prolongation in progression-free survival with taselisib,^6^ and SOLAR-1 (ClinicalTrials.gov identifier: NCT02437318) demonstrated a clinically meaningful improvement in progression-free survival with alpelisib.^7^ This led to the approval of alpelisib in combination with fulvestrant in *PIK3CA*-mutant, hormone receptor–positive (HR+), metastatic BC.

Activation of the PI3K pathway is frequent in BC brain metastases, as evidenced by observation of AKT and S6 phosphorylation and loss of *PTEN*.^8^ Recent data suggest *PIK3CA*-activating mutations may be associated with an increased risk of CNS metastases in patients with ER+/HER2− disease.^9^ In 307 patients with ER+/HER2− metastatic disease, brain metastases were significantly more common in patients with *PIK3CA* mutations (30.8% *v* 17.1%; *P* = .0049). Treatment of brain metastases from ER+ BC remains difficult. A recent retrospective analysis found a clear association of improved survival with continuation of endocrine therapy upon diagnosis of brain metastases.^10^ Remarkably, the CDK4/6-inhibitor abemaciclib had a clinical benefit rate of 25%.^11^ Preclinical models of BC brain metastases suggest PI3K pathway inhibition may be effective for treatment of brain metastases.^12^ Notably, both SANDPIPER and SOLAR-1 excluded patients with untreated or active CNS metastases^6,7^; however, the precursor to alpelisib, buparlisib, did have brain penetration, which was thought to be the cause for the higher incidence of mood disorders.^13^ An increase in depression has distinctly not been seen with the α-specific PI3K-inhibitor alpelisib and, in preclinical animal models with intact blood-brain barrier, no significant distribution into the brain was seen (unpublished data). Thus, the activity of alpelisib in brain metastases is unknown.

**FIG 1.**
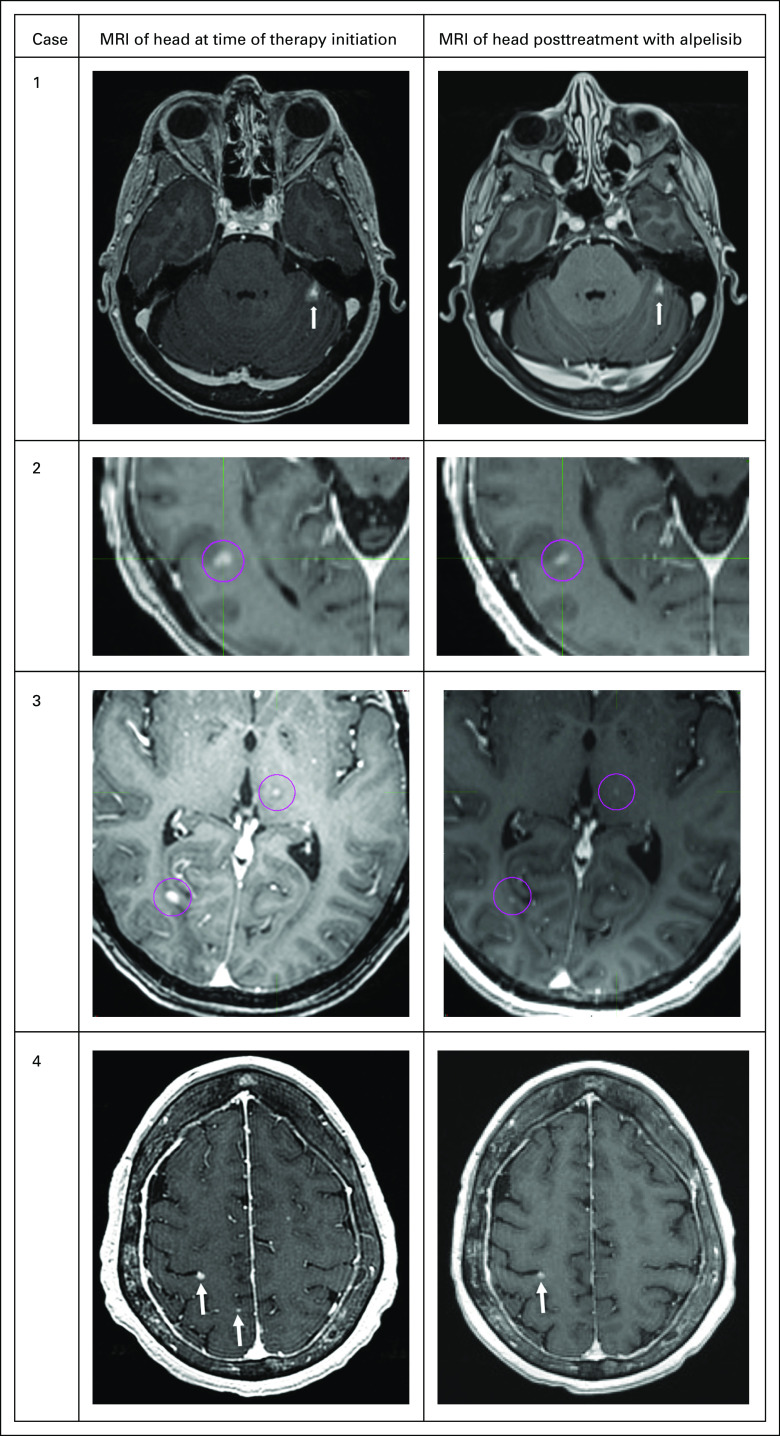
CNS response measured by magnetic resonance imaging (MRI) of the brain. Pink circles and white arrows represent metastatic disease from breast cancer.

Herein, we report a case series of 4 patients with ER+/progesterone receptor–positive (PR+)/HER2− metastatic BC with progressive brain metastases (Fig 1) treated with alpelisib. All patients provided consent to publish their information and images.

## CASE 1

A 55-year-old woman presented with a large breast ulceration, biopsy specimen–diagnosed invasive ductal carcinoma (IDC), grade 3, ER+/PR+/HER2−. Computed tomography (CT) scan revealed pulmonary nodules, osseous lesions, and hypodense lesions within the right hepatic lobe. Brain magnetic resonance imaging (MRI) showed a 10-mm mass in the left cerebellar hemisphere; this was treated with stereotactic radiosurgery with initial shrinkage to 8 mm and stabilization on follow-up. Tumor sequencing showed an activating *PIK3CA* mutation *H1047R* and amplification of *PIK3C2B*. Disease progressed after 4 months of treatment with fulvestrant and palbociclib. Brain MRI showed an increase of the left cerebellar lesion to 12 mm, judged by neuroradiology and radiation oncology to be more compatible with progression than with radiation-induced tissue necrosis. Palbociclib was switched for alpelisib (300 mg daily) with continuation of fulvestrant and zoledronic acid. The patient’s body mass index (BMI) was 25 (calculated as kilograms divided by square of height in meters) and Eastern Cooperative Oncology Group (ECOG) performance status (PS) score of 0. Subsequently, the patient’s chest wound started to heal with epithelization and closure of the wound. Brain MRI after 6 weeks showed a reduction in the left cerebellar lesion to 9 mm (62% reduction of bidimensional areas, 35% reduction of sum of longest distances). Follow-up MRI 2 months later revealed stability of the CNS lesion. Subsequent brain MRI at 3, 4, and 6 months showed stable disease without changes in measurements. This was compatible with a partial response per Response Assessment in Neuro-Oncology Brain Metastases (RANO-BM) criteria.^14^

## CASE 2

A 71-year-old woman was diagnosed in 2010 with pT2N0 IDC of the right breast, grade 3, ER+/PR+/HER2−. She was treated with breast-conserving surgery followed by adjuvant doxorubicin plus cyclophosphamide therapy and radiation. She completed 5 years of adjuvant tamoxifen in 2016. In 2017, she had a recurrence in the right breast, pleural effusion, and bone metastases and was treated with multiple regimens: letrozole and palbociclib, fulvestrant and palbociclib, and capecitabine and paclitaxel. Brain MRI showed multiple (> 10) subcentimeter metastases. Testing of primary tumor had demonstrated a *PIK3CA* *H1047R* mutation and *TP53* mutation (*H179Q*). Because the patient was asymptomatic from the brain metastases, whole-brain radiotherapy (WBRT) was deferred and she was administered treatment with fulvestrant plus alpelisib (300 mg daily). Her BMI was 23 and ECOG PS score was 0. Brain MRI 4 weeks later demonstrated minor regressions of nonmeasurable, subcentimeter brain metastases without new or progressive lesions. Brain MRI performed 10 weeks after initiation of therapy demonstrated stability of CNS lesions; however, CT scan demonstrated progression of liver metastases, which prompted change of therapy to eribulin. A liquid biopsy specimen at that time was notable for continued presence of *PIK3CA* *H1047R* and *TP53* *H179Q*, and an acquired *ESR1* (*Y537N*) mutation (minor allele frequency, 1.4%).

## CASE 3

A 55-year-old woman was diagnosed in 2009 with pT2N2 IDC of the left breast, grade 2, ER+/PR+/HER2−. She was treated with mastectomy followed by dose-dense doxorubicin plus cyclophosphamide and paclitaxel followed by radiation. She completed 5 years of tamoxifen therapy and then letrozole. Workup for hip pain in 2015 demonstrated metastatic ER+/PR−/HER2− BC. She was serially treated with fulvestrant and palbociclib, radiation to the base of skull to treat cranial nerve symptoms, and with exemestane and everolimus, capecitabine, and abemaciclib. For the first time, brain MRI demonstrated multiple parenchymal metastases and she was treated with hippocampal-sparing WBRT. Subsequently, treatment was changed from abemaciclib to liposomal doxorubicin for progression in the brain, followed by gemcitabine. She continued to have progressive CNS parenchymal disease. Testing of primary breast tumor demonstrated a *PIK3CA* *E545K* mutation. She was administered treatment with fulvestrant and alpelisib (300 mg); her BMI was 23 and ECOG PS score was 0. Brain MRI 6 weeks later demonstrated a 14% reduction in the sum of longest distances of measurable brain metastases (24% reduction in bidimensional areas) and regressions of nonmeasurable brain metastases. Repeated brain MRI at the 4-month mark showed mixed response, still compatible with stable disease by RANO-BM criteria,^14^ and treatment was continued. The most recent brain MRI at the 6-month mark revealed progressive parenchymal disease requiring change of therapy.

## CASE 4

**FIG. A1.**
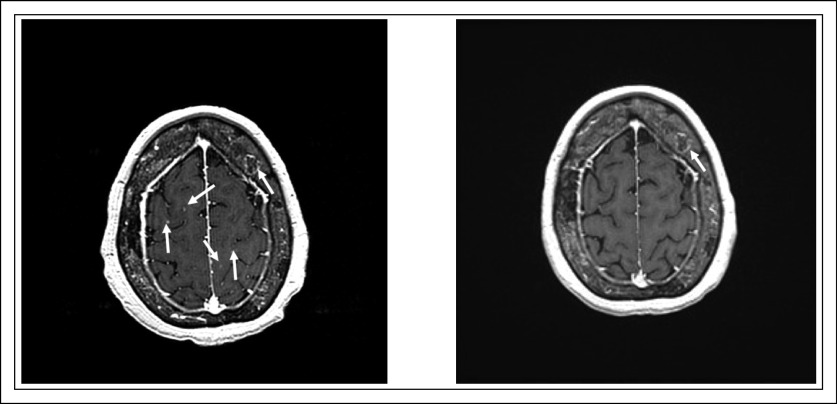
Additional cuts showing further evidence of response in case 4. Left image was taken before treatment with alpelisib and exemestane; the right image was taken posttreatment. White arrows are directed to lesions, most of which were no longer visible after treatment.

A 70-year-old woman was diagnosed in 2013 with T1N1 IDC of the right breast, grade 3, ER+/PR+/HER2−. She was treated with breast-conserving surgery, adjuvant docetaxel plus cyclophosphamide, radiation, and anastrozole. In 2018, metastatic disease developed to the lung, liver, and bone while she received adjuvant anastrozole. Examination of a liver biopsy specimen confirmed recurrent metastatic ER+/PR−/HER2− BC. Brain MRI performed for dizziness revealed millimeter-size lesions in the right parietal and left posterior frontal lobes and the left frontal lobe. The patient received stereotactic radiation followed by therapy with fulvestrant, palbociclib, and denosumab. At 3 months, capecitabine was started to treat progressing liver metastases. Follow-up MRI demonstrated at least 15 new intraparenchymal lesions and several progressing dural-based lesions, so the patient proceeded to undergo WBRT and restarted capecitabine therapy. Restaging 6 weeks later revealed disease progression in the lungs, liver, and brain parenchyma. Examination of a liquid biopsy specimen revealed multiple PI3K mutations (*PIK3CA*: *H1047R*, *E81K*, and *E563K*) and she was administered alpelisib (300 mg) with exemestane. Her BMI was 22 and ECOG PS score was 1. The patient’s pretreatment HbA1c value was 6.3%; despite avoiding sugars, she required admission for hyperglycemia after starting alpelisib treatment and glucose levels have been difficult to control. Restaging after 6 weeks of therapy revealed substantial disease regression in the lungs and liver as well as interval resolution of punctate cerebellar and cerebral metastasis and reduction of a dural metastasis (Appendix Fig A1), compatible with stable disease per RANO-BM criteria.^14^ Repeated brain MRI at the 3- and 5-month marks showed stable disease.

## DISCUSSION

Activating *PIK3CA* mutations occur early in breast carcinogenesis and are typically not lost or acquired during clonal evolution in later stages of the disease—features that suggest these are driver mutations. Table 1 provides a summary of mutational profile and previous treatments. Cases 1 and 2 harbored the activating mutation *PIK3CA* *H1047R* in the coding exon 20, a kinase-activating mutation and the most common mutation in BC.^14^ Case 1 also had *PIK3C2B* amplification, which occurs in 13% to 25% of patients.^15^ *PIK3C2B* is a class II PI3-kinase.^16^ Whether its amplification in conjunction with *PIK3CA* mutation deepens or attenuates PI3K-pathway dependence of cancer and whether *PIK3C2B*’s kinase activity is inhibited by alpelisib is unknown. However, understanding this relationship appears to be important because activating *PIK3CA* mutations and copy number gain of *PIK3C2B* do co-occur in BC.^15^ Case 3 harbored the *PIK3CA* E545K mutation, affecting the helical domain of p110-α that activates signaling because of its detachment from the inhibitory p85 subunit of PI3K.^17^ Helical domain mutations are the second most frequent in BC, with an incidence of 6.4%.^14^ Case 4 had 3 mutations in *PIK3CA*; E81K and *H1047R* are activating mutations and E563K is novel. This combination of a major (*H1047R*) and a minor (E81K) *PIK3CA* mutation is thought to amplify PI3K signaling and predict for responsiveness to PI3K inhibition.^18^

**TABLE 1.**
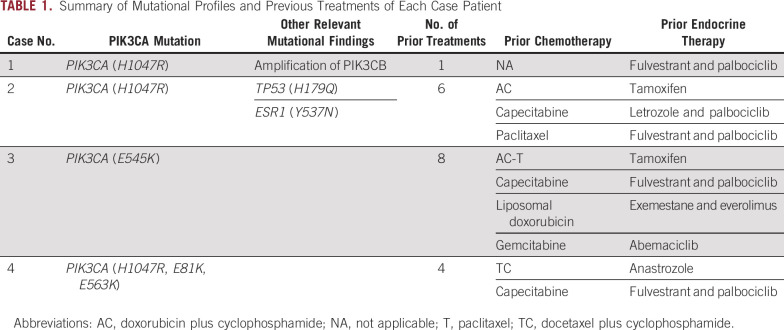
Summary of Mutational Profiles and Previous Treatments of Each Case Patient

This patient had a comutation of *PIK3CA* mutations and *ESR1*. These are uncommon and accounted for 10% in a small study with 86 ER+ endocrine therapy–resistant metastatic BC.^19,20^ Our query of the 453 samples of metastatic BC available via cBioPortal (Memorial Sloan Kettering Cancer Center, New York, NY), indicated a tendency for co-occurrence of these 2 mutations (log_2_ odds ratio, 1.518; *P* = .003).^21^ Data specific to brain metastasis were not available for such analysis. This case was also notable for discordance at the time of progression: liver lesions progressed whereas brain lesions did not, possibly a result of the outgrowth of clones with alterations that convey resistance such as loss of *PTEN*.^22^

In the SOLAR-1 study, the majority of *PIK3CA* mutations were activating mutations in p110α *H1047(X)* (54%), and although the hazard ratio was similarly favorable for all mutations, the CIs in the *H1047(X)* group were the most narrow, meaning these patients most reliably responded to alpelisib.^7^ Consistently, 75% of patients in our series also had a *H1047(X)* mutation.

A recent study showed that *PIK3CA*-mutant BC not only has a higher probability to metastasize to the brain than nonmutant BC (31% *v* 17%) but also has shorter median overall survival after the diagnosis of CNS metastasis (0.5 *v* 1.1 year).^9^ The high frequency of brain metastases and their poor prognosis raise the question whether PI3K inhibitors can be beneficial for patients with active brain metastases. The earlier pan-PI3K inhibitor buparlisib was tested in 4 patients with treatment-refractory CNS lymphoma, 1 of whom achieved a partial remission.^23^ In animal models, systemically administered buparlisib showed activity against BC brain metastases.^24-26^ Patients with active brain metastases were excluded from the pivotal SOLAR-1 and SANDPIPER studies.^6,7^

Conclusions from our observations are limited by the small number of patients selected on the basis of their surprising response. The RANO-BM criteria consider lesions < 10 mm as nonmeasurable.^14^ Here, we show examples with resolution of small lesions, which are clinically meaningful but classified as stable disease per the criteria. Nevertheless, our observations suggest the presence of CNS metastases may lead to sufficient disruption of the blood-brain barrier to enable drug activity in the CNS. Additional investigation to prospectively evaluate alpelisib in patients with BC and CNS involvement may be justified.

In conclusion, we have described cases of regression or stabilization of progressive CNS lesions in patients with HR+/HER2− metastatic BC treated with the PI3K inhibitor alpelisib. On the basis of these observations, we believe additional clinical investigation of PI3K inhibition in patients with brain metastases is warranted.
